# Case of Man vs. Monkeys, Dogs Et. Al.

**Published:** 1884-03-20

**Authors:** E. Van Goidtsnoven

**Affiliations:** Georgia


### CASE OF MAN VS. MONKEYS, DOGS ET. AL.

BY E. VAN GOIDTSNOVEN, M.D., OF GEORGIA.

But a short time ago the bill posters of Paris were busy spreading on the walls of the great French metropolis a large yellow placard representing two monstrosities in human form, whose faces were entirely covered with hair, and on this account, although very improperly, as will be presently shown, they were heralded as "The Man Dog and His Son."

The fact is, these two phenomena have nothing of the dog, not even its intellect. Indeed, intellect seems to be null in them or, at least, considerably obtuse. A veil—thick and, I might say, impervious—covers it, screens it from scrutiny as effectively as does the immeasurably long hair hide from view the features of either individual.

A particularity which distinguishes them from other men is the extraordinary length of the facial hair. The beard, long, silky and glossy, ascends up to the eyes, and the hair encroaches so low on the forehead as to mingle with the eyebrows. To this add an incomplete development of the jawbone, which presents only ten teeth in the man and eight in the boy, and you will have all the characteristics whose sum total form these strange anomalies. The main one, the most striking, and, if I may be permitted to say it, the most specific, consists simply in the superabundance and excessive length of the hair, which, although covering the whole body, can only be seen on a few selected and determined places.

"The Man Dog and his Son"—I shall name them so, since they are introduced to us under that cognomen—form, therefore, neither a distinct species nor a distinct race. Their shaggy aspect in no way should constitute a very remarkable monstrosity any more than the total absence of beard ordinarily seems to draw particular attention. The man presents nothing strange beyond his beard, hair and teeth. He is of medium stature, and well proportioned. His broad shoulders and slightly bent dorsum indicate a great muscular strength. His look bespeaks no intellect. The color of his hair is of a dark brown. As to the boy, he is almost an albino, and, on this account, more interesting than the father. Such are the two individuals torn, as it were, from the dense forests of the government of Kostroma, Russia.

There exists a race of men remarkable in many respects, and particularly on account of the extraordinary development of their pilous system. I refer to the true aborigines of Japan, called Ainos, the genuine autochthous of the Empire of the Rising Sun. Once the proud possessors of the soil, they have been conquered, evicted and driven off by the Japanese, the ruling race of to-day. The Ainos, now banished in the islands of Yesso, Karafto, Kuriles and a portion of Tunkuse Tartary, still remember their former prestige and power. If the Japanese consider them as uncouth savages, unacquainted with, or rather indifferent to the usages, customs, manners and modus vivendi of Japan, the Ainos, in their turn, look upon the Japanese as intruders and pirates, living on plunder and ill-gotten possessions. In proof thereof I will cite the following epigram of Abeno Moneto, who, having been defeated by the troops of the Mikado and brought a captive to the Japanese Capital, remarked to the sneering courtiers, as he pointed out to them the gorgeous display of some Medlars blooming near by: "There I recognize the blossoming gems of my country, the flower of the Medlar! But, you men of the court, how do you name them?" The Ainos entertain no feeling but that of utter contempt for their conquerors, who are, as it were, beardless. Brave, robust, strong and industrious, they cling to their soil with a truly heroic tenacity, and culrivate it *cum amore*. They are of a peaceable and complying, though not over-confiding temper. In their language, which is not the Japanese, their name signifies *man par excellence*. Their race is evidently a primitive one, and may be said to be of no mixed nor cross blood, free from any foreign streak. The color of their hair is either black or fawn, that of their skin red—not unlike that of the Red Skins of America, which some authors contend was originally peopled by them.

The shaggy tribes have in all times, and especially in antiquity, enjoyed a high reputation for strength, courage and bravery. Homer, singing in his immortal Iliad the bloody quarrels and strifes of the wild ancestors of the Hellanes, takes special care in dwelling upon the fact that Achilles, Agamemnon and other famous warriors had shaggy breasts, thus producing an incontestable proof of corporal strength and superior physical attainment. Isaiah, in his prediction of the fall and ruin of Babylon, says that she will fall a prey to the dragons and *satyrs*. who shall take their sport therein. According to Mythology and Webster's definition, a *satyr* is a sylvan deity, a demi-god, a monster, part man and part goat, and characterized by riotous merriment and lasciviousness. As there is no myth, neither in nature nor in Divine revelation, there can be but one rational interpretation of the word *satyr* in this instance; that is, man with long hair or wool and rugged, shaggy skin, like the Ainos.

Western Europe was once governed under the sway of the powerful Tartarian Ainos. Attila was the son of an Ainos, and the Huns were, in all probability, the descendants of that old race, which is still extant and lives in Japan. They were, doubtless, driven from their native land by either famine, pestilence or the incursion of a race superior to theirs in strength, number or power. History tells us that they rushed like a torrent on Europe, which they overran and devastated in every direction until they met with defeat and death in the Catalonian plains of France. According to tradition, Sweyne, King of Denmark, was of the same origin.

Those men with bodies and faces disappearing, so to speak, under a thick layer of hair naturally suggest the idea of associating them with monkeys, to whom they would seem to be closely related. Such, indeed, was the estimation antiquity set upon them. The Ramayana, an Indian poem which relates their conquest of India at a very remote period, describes them as true monkeys. The strange aspect of the conqueror, together with the hatred they inspired in the conquered, more than likely contributed to stamp such a prejudice within the public mind of those days and force it to accept the myth with as implicit a faith as though it were a dogmatic truth.

But all these are merely idle speculations, and simply amount to naught. Man, no matter how material the deformity under which he presents himself, does not descend from the monkey any more than the monkey descends from man. In vain do Darwin and his followers endeavor, in their study of human origin, to assign to man and the monkey a common parentage, a common ancestry; they cannot help their opinion being received only as a purely gratuitous hypothesis, which no fact has hitherto either corroborated or confirmed. But the objection may arise: "What of the Niams-Niams, or negroes with caudal appendices, said to have been found in Africa? To this I will simply reply that those monkeys whom Nature has brought nearest to man have no tails, and, consequently, should rank higher than the Niams-Niams in the scale of intellectuality. They should be more civilized than the Niams-Niams, and, like them, possess—but in a higher degree—the power of speech! And yet such is not the case.

But do the Niams-Niams, such as have been described a few years ago, really exist? Is it true that a whole tribe of Negroes has been vouchsafed the glorious privilege of a prolongation of the spinal column in the form of a tail?

Numerous testimonies of the most positive character and the writings of credible observers have been adduced in support of an affirmative answer. True, the majority were not eye-witnesses, but gave a faithful account of all the minute details that were given them in the language of asseveration. One instance represents an Abyssinian priest, a man free from credulity and remarkable for his great learning and depth of thought, giving the minutest details concerning the tail, its situation, form, length, the fawn color of the hair covering it, etc., etc.; not an item remains untold, and every point seems to be obviously explained and irrefutably established. Another instance is that of a distinguished traveler obtaining from a trustworthy negro valet a full and complete unraveling of the whole mystery. Moreover, it is contended that the existence of men with tails, called Niams-Niams, is so well known and affirmed in Egypt and Turkey that the populations of those countries express their wonder at its being disputed in nations claiming to be civilized. "Everybody in Turkey believes it; why do you not believe it, too?" Such is the remark made to any doubtful Westerner. Orientals are prone to be credulous in matters savoring of the marvellous. The rest of the world is not apt to accept with an implicit faith those prodigious or fabulous statements. Solid reasons and positive proofs are absolutely necessary to convince a respectable portion of mankind. The latter, like St. Thomas, wish to see and to touch, and even then are not fully convinced.

The following was written by a physician attending the hospitals of Constantinople:

"In 1852 I saw, for the first time, a negress with a tail. Struck with this phenomenon, I questioned her master, who was a slave-trader. He informed me that there exists a tribe called Niam-Niam, living in the very depths of Africa. Every member of that tribe bears the caudal appendix; and, as the Oriental imagination is very prone to exaggeration, he assured me that a tail two feet in length is not a very rare thing to behold. The one I saw was smooth and hairless, two inches in length and tapered down to a point. The color of this woman was jet black, her hair very thick, matted and woolly. Her teeth were purely white, coarse and set in alveoli strongly projecting outward. The four incisors had been filed. Her eyes were bloodshot. She fed on raw meat. Her garments greatly annoyed her. Her intellect seemed to be at par with that of the members of her tribe.

"Her master had been trying to sell her for six months, but could find no purchaser, notwithstanding he proposed to dispose of her at a low price. The cause of the disgust she inspired did not lie in her tail, but in her appetite and avowed cravings for human flesh. Her people live on the flesh of prisoners captured in their constant warfares with neighboring tribes.

"As soon as one of them dies, the relatives, instead of proceeding to inhumation, carve the remains and feast thereon. Of course there are no cemeteries in that region. They do not all lead a wandering life. Many of them dwell in huts constructed out of forest growth. They are skilled in the manufacture of war and agricultural implements. They cultivate Indian corn and rye, and raise cattle. The Niams-Niams speak a language of their own, the composition of which is very primitive, interspersed with a few Arabic words.

"They live in complete nudity, and only seek to gratify their sensual appetites. The strongest amongst them is the de facto and de jure chief of the tribe. He alone divides and distributes the spoils. It is not known whether they have a religion. It is hardly probable that they have, when one considers the facility with which they embrace any creed that is preached to them.

"To tame them completely is a most arduous task. Their instinct impels them to the devouring of human flesh, and many instances are cited of slaves having massacred and devoured children entrusted to their care for safe-keeping.

"Last year I saw a man of this tribe with a tail an inch and a half long, covered with a few hairs. He appeared to be thirty-five years of age, robust, well-proportioned, jet black, with the same peculiar conformation of the jaw as above cited, namely, with alveolæ projecting outward. It is customary with those people to file the four incisors in order to lessen the masticating power."

Other writers, like the physician of Constantinople, affirm that they have seen with their own eyes and touched with their own hands this strange appendix. Although their statements are at variance as to the thickness, length and color of the caudal hair, yet they are unanimous in affirming the existence of the appendix and its firm adhesion to the spinal column. Hence, the natural conclusion that there must, necessarily, exist a negro tribe wherein the tail is an hereditary appendage. Such is not the case. Further inquiry and developments have proved this to be an idle inference. The Niams-Niams are born without a tail. That which they bear is but the result of a graft skillfully inserted. Hence, this diversity and variety of tails above mentioned.

With the Niams-Niams the tail is a distinctive mark of high rank, and entitles its proud possessor to the right of proclaiming that he is of free and noble birth or, in Virginian parlance, a lineal descendant of one of the F. F. N. N. With them, therefore, the tail is synonymous with heraldry—a true coat of arms.

"Honi soit qui mal y pense!" Fashion, customs and manners vary with every people!

